# Effect of environmental NGOs on human health in China: An empirical analysis

**DOI:** 10.1371/journal.pone.0284468

**Published:** 2023-05-17

**Authors:** Wenxin Wang, Muhammad Hafeez, Ziyu Guo, Muhammad Yasin Zia, Raufhon Salahodjaev, Iftikhar Ali

**Affiliations:** 1 School of Public Health, Shantou University/Institute of Local Government Development, Shantou University, Shan-Tou, 515063, P.R. China; 2 Institute of Local Government Development, Shan‐Tou, 515063, People’s Republic of China; 3 Institute of Business Management Sciences, University of Agriculture, Faisalabad, Pakistan; 4 Center for Disease Control and prevention, Bao’an District, Shenzhen City, Guangdong Province, China; 5 Department of Mathematical Methods in Economics, Tashkent State University of Economics, Tashkent, Uzbekistan; 6 Department of Agronomy, University of Agriculture, Faisalabad, Pakistan; Huanggang Normal University, CHINA

## Abstract

The emergence of environmental nongovernmental organizations (ENGOs) has proved beneficial in improving environmental quality and related health issues. Therefore, this study attempts to investigate the impact of ENGO on human health in China from 1995 to 2020. To investigate the relationship between the variables, we have employed the ARDL model. The ARDL model results demonstrate that the long-run impact of ENGO is negative on infant mortality and death rate, meaning that an increase in the proportion of ENGOs in China considerably decreases infant mortality and death rate. On the other hand, ENGOs have a favorable influence on life expectancy in China, demonstrating ENGOs’ supporting role in raising birth life expectancy. In the short run, estimates of ENGOs have no substantial influence on newborn mortality and death rates in China, whereas ENGOs have a positive and significant impact on life expectancy. These results imply that ENGOs help improves people’s health status in China, which is also supported by the rise in GDP, technology, and health expenditures. The causal analysis confirms the bi-directional causal link between ENGO and IMR and ENGO and LE, while the unidirectional causal link runs from ENGO to DR. The results of the study provide insights into the impact of environmental NGOs on human health in China and may help guide policies aimed at improving public health outcomes through environmental protection efforts.

## Introduction

Global warming, greenhouse gas (GHG) emissions, and environmental destruction have risen to the forefront of international environmental agendas in recent years. One of the biggest environmental problems in the world today is the rising concentrations of carbon dioxide (CO2) and other GHGs in the air. More than 60 per cent of the greenhouse effect may be attributed to carbon dioxide, which plays a pivotal role in amplifying the influence of the other GHGs [1,2]. As a result, officials need to take a hard look at CO2. Given that subsequent generations will need the environmental resources for their survival and prosperity, environmental sustainability is among mankind’s most pressing challenges [3,4]. Consequently, it is our duty to safeguard the environment for coming generations.

The dispute over the relationship between healthcare, CO2 emissions, and human development index (HDI) has been a popular issue in the financial literature for both developed and emerging nations during the last 20 years. Authorities, legislators, academics, and economists are all working to guarantee that mankind is living in a safe, healthy, clean environment today [5]. All nations depend critically on government spending, particularly in the area of healthcare. Additionally, global warming is the greatest problem of our day and a danger to natural heritage, safety, and affluence [6]. The Inter-Governmental Panel on Climate Change (IPCC) asserted that CO2 emissions were the primary factor responsible for global warming in 2013. The "Kyoto Protocol," signed by more than 100 nations in 1997, set a goal of lowering CO2 emissions to protect advanced nations from the harmful consequences of climate change [7,8]. Traditionally, emerging nations did not give importance to combating climate change; however, due to their fast economic development, these nations now show a major worry about doing so [9,10]. Furthermore, Wei et al. [11] demonstrated that in the context of emerging nations, a rise in economic development and energy demand is highly associated with CO2 emissions, which are hazardous to the ecosystem.

Limited research in recent times has demonstrated a positive correlation between medical costs and carbon dioxide emissions [12]. Given the negative effects of pollutant emissions on public health, quantifying the impact of CO2 emissions on healthcare costs is an intriguing field of study [13]. The correlation between carbon footprint and health risks was originally shown by Dong et al. [14]. Through the use of the "high-resolution model", he discovered that CO2 contributes to an upsurge in mortality in the United States because of deteriorating environmental quality. The research also shows that CO2 discharges increase surface ozone and particle matter (PM), which raises illness and mortality rates. Therefore, CO2 emissions may raise the costs associated with maintaining a healthy lifestyle for individuals. Cianconi et al. [15] also investigate the relationship between CO2 concentration and mental health. The empirical results imply that office employee who is less subjected to CO2 perform better cognitively than their peers. In a similar vein, Chaabouni and Saidi’s [5] research shows that GHG emissions, particularly CO2 emissions, have a detrimental impact on human health. For instance, Khoshnevis Yazdi & Khanalizadeh [16] demonstrated that both CO2 and carbon monoxide had a positive connection with healthcare spending in Iran.

With regard to ecologically friendly activities, nongovernmental organizations (NGOs) look to have a more stable role in global governance [17]. United Nations charter has historically acknowledged the significance of NGOs in environmental preservation. "Our Common Future", a document by the world commission on environment and development (WECD), urged governments to "recognize and extend NGOs’ right to know and have access to information on the environment and natural resources; their right to be consulted, and participate in decision-making on activities likely to have a significant impact on the environment; and their right to legal remedies and redress when their health or environment may be seriously affected". Even though NGOs play an essential role in environmental protection, their impact is seldom assessed in depth in contrast to other government agencies [18]. Because of the unexpected increase in ecological activity over the last several years, the study is pulled off a little. In principle, the term "NGO" can be utilized to refer to a variety of climate actors, from grassroots organizations of local environmental activists or pollution victims to large, well-established, highly skilled international corporations that are similar to supervisory bodies but not publicly accountable [19]. Health research is a multi-step process that requires not just the creation of new information but also the prioritization and dissemination of that information to be successful. NGOs are often seen as valuable partners in this endeavor [20]. Nongovernmental organizations (NGOs) have made invaluable discoveries by funding important and useful research [21,22].

China is the world’s largest emitter of carbon emissions [23]. Therefore, the negative effects of pollution on Chinese citizens’ health have come into sharper focus in recent times. Despite the Chinese administration’s increasing spending on ecological sustainability and the subsequent decrease in the production of certain pollutants, severe issues, including air, water, and soil contamination, persist, with grave consequences for human health [24]. Less than 1% of China’s 500 biggest cities satisfy air quality criteria suggested by the WHO, as well as seven of the globe’s most contaminated cities are in China, according to a new analysis by the Asian Development Bank and Tsinghua University. In China, the role of civil society is slowly but surely increasing in policy-making regarding the environment [25]. ENGOs are not only actively taking part in improving environmental quality but also trying to mitigate related health problems. However, there is a dearth of empirical evidence when it comes to the relationship between ENGOs and human health in China.

This study explores the impact of environmental NGOs on human health in the case of China using the ARDL approach. The topic of the impact of environmental NGOs on human health is relevant for several reasons. Firstly, environmental NGOs play a crucial role in raising awareness about environmental issues that have a direct impact on human health. By promoting environmental protection measures and highlighting the potential health risks associated with environmental pollution, these organizations can help individuals and communities take action to protect their health. Secondly, environmental NGOs often conduct research and gather data on environmental health hazards, which can inform policy decisions and help shape regulations that protect human health. They may also provide education and training to individuals and communities on how to mitigate the negative impacts of environmental pollution on their health.

There are significant gaps in the existing body of studies in various ways. Firstly, previous research on human health in China did not consider the impact of environmental NGOs [26,27]. However, environmental NGOs are a key factor in explaining healthcare services. Secondly, prior studies relied on outdated techniques for estimation, which can lead to biased results. This study contributes new empirical evidence to the field of health economics. Unlike previous research, it examines the impact of NGOs on human health. Moreover, this study utilizes the ARDL approach to examine the long-run and short-run dynamics of environmental NGOs on human health in conjunction with health expenditures, GDP, and technology. The ARDL methodology is a useful tool for econometric analysis, particularly when dealing with mixed-order integration and non-stationary time series data. It allows for the estimation of long-run relationships between variables, including short-term and long-term effects, and it can be used to test for the presence of cointegration between the variables. The ARDL methodology is a useful tool for investigating the relationship between environmental NGOs and human health outcomes. It allows us to estimate the long-run relationship between these variables, while controlling for other factors that might influence human health, and to investigate the short-term and long-term effects of environmental NGOs on human health. Despite the importance of the topic, a comprehensive and up-to-date analysis of China is currently lacking, and a study that can measure the influence of environmental NGOs on human health in China is necessary, given the contentious nature of the issue. Lastly, its findings will be useful for environmentalists, government officials, health researchers, policymakers, and the general public in evaluating and understanding how environmental NGOs impact human health in the long-run and short-run.

Studying the impact of environmental NGOs on human health can enrich the knowledge of the scientific community in several ways. Firstly, environmental NGOs often focus on protecting and preserving the environment, which can have a direct impact on human health. Studying the impact of these NGOs can help policymakers to understand the link between environmental degradation and human health. Secondly, environmental NGOs may employ a variety of strategies to raise public awareness about environmental issues and promote public health. Thirdly, research on the impact of environmental NGOs can inform the development of new interventions and policies aimed at protecting public health. For example, if a study finds that a particular type of intervention used by environmental NGOs is effective; policymakers may consider implementing similar measures on a larger scale. Lastly, studying the impact of environmental NGOs on human health requires collaboration between researchers in different disciplines, such as environmental science, public health, and social science. Such collaborations can help bridge the gap between different fields of study and promote a more comprehensive understanding of the complex relationship between the environment and human health.

### Literature review

Environmental NGOs have emerged as important players in addressing environmental issues globally. These organizations often play a significant role in advocating for the improvement of human health by addressing environmental pollution, which can have negative effects on public health. This literature review aims to examine the effect of environmental NGOs on human health in China, focusing on the studies that explore the impact of these organizations on improving health quality. China has been facing severe air pollution and water pollution problems due to industrialization and urbanization [28]. In recent years, several studies have explored the effect of environmental NGOs on improving air quality and water quality in China. Wang et al. [29] investigated the effect of environmental NGOs on reducing PM2.5 pollution in China. The study found that the increased presence of environmental NGOs is associated with a reduction in PM2.5 concentration, which is a critical pollutant that contributes to respiratory and cardiovascular diseases. Li et al. [18] investigated the impact of environmental NGOs on water quality improvement in China. The study found that environmental NGOs have played a critical role in promoting public awareness about water pollution and advocating for better water quality standards and have contributed to better public health outcomes. A study by Vassanadumrongdee & Kittipongvises [30] investigated the role of environmental NGOs in promoting human health in Thailand. The study found that environmental NGOs have played a significant role in promoting human health. A study by Gulzar & Henry [31] investigated the relationship between participation in environmental NGOs and health outcomes in the Pakistan. The study found that people who participated in environmental NGOs have experienced good quality health. Another study by Fasoli [32] explored the relationship between environmental NGOs and health in the Netherlands. The study found that people who are members of environmental NGOs reported good quality of health than those who are not members.

After conducting a thorough search of the existing literature, it appears that there is no direct research on the effect of environmental NGOs on life expectancy. However, there is some related research on the broader impact of environmental factors on health outcomes and the role of NGOs in promoting environmental protection. A study conducted by the World Health Organization (WHO) in 2016 found that environmental risks contribute to about 23% of global deaths, which corresponds to 12.6 million deaths per year [33]. These risks include air pollution, unsafe water, lack of sanitation, and inadequate hygiene. Therefore, it is clear that environmental factors have a significant impact on health outcomes, including life expectancy. Environmental NGOs play a vital role in promoting environmental protection by advocating for policy changes, conducting research, and raising public awareness about environmental issues [34]. There is some evidence to suggest that the work of NGOs has contributed to positive environmental changes that may have indirect effects on life expectancy [35]. For example, a study by Costello et al. [36] found that conservation efforts led by NGOs have contributed to a reduction in deforestation in tropical regions, which can help to mitigate the impacts of climate change and improve health quality.

Another study by Tu et al. [37] examined the impact of environmental NGOs on water quality in developing countries. The authors found that NGOs that work to improve access to clean water and sanitation have contributed to a significant reduction in diarrheal diseases, which are a major cause of mortality in these regions. The literature suggests that environmental NGOs can have an important indirect effect on life expectancy by promoting environmental protection and improving access to clean water and air [38]. However, more research is needed to fully understand the specific impact of these organizations on life expectancy and to explore potential causal mechanisms.

After conducting a comprehensive search of the existing literature, it appears that there is some evidence to suggest that environmental NGOs may have a positive effect on reducing mortality rates, particularly in developing countries. One study conducted by Zhang et al. [39] found that environmental NGOs can help to reduce mortality rates in Chinese cities by improving air quality. The authors found that cities with more active environmental NGOs had lower concentrations of particulate matter and sulfur dioxide in the air, which are associated with increased mortality rates. They concluded that environmental NGOs can play an important role in improving air quality and reducing mortality rates. Another study by Dagestani et al. [40] examined the impact of environmental NGOs on malaria incidence and mortality rates in Sub-Saharan Africa. The authors found that environmental NGOs that work on initiatives to reduce deforestation and increase access to clean water have contributed to a reduction in the prevalence of malaria and the mortality rate associated with the disease. They concluded that the work of environmental NGOs in these areas has the potential to contribute to improved health outcomes in Sub-Saharan Africa. In addition, a systematic review by Khomsi et al. [41] found that environmental interventions, including those implemented by NGOs, have the potential to improve health outcomes and reduce mortality rates in low- and middle-income countries.

In conclusion, the literature suggests that environmental NGOs may have a positive impact on human health, particularly in developing countries. However, further research is needed to fully understand the mechanisms through which environmental NGOs affect health outcomes and to identify the most effective interventions. Additionally, NGOs must address the challenges and limitations they face in their work to maximize their impact on health outcomes.

### Models, methodology, and data

The impact of environmental NGOs on human health is multifaceted and can be both direct and indirect. By promoting environmental sustainability and protecting natural resources, environmental NGOs can help to create healthier and more equitable communities. Environmental NGOs can advocate for policies that promote clean air and water, which can have significant impacts on human health [42]. Similarly, environmental NGOs can provide information to communities about environmental health risks and how to reduce exposure to these risks [37]. However, environmental NGOs can work to address environmental injustices, which can have significant impacts on human health. Our study is based on the social-ecological theory and developed by Janssen & De Vries [43]. This model proposes that human health is influenced by a complex interplay of social, environmental, and economic factors. The model suggests that environmental NGOs play a critical role in promoting health by advocating for policies and programs that address environmental issues and by empowering individuals and communities to take action to protect the environment. Following former literature by Shandra et al. [44] and Piotrowicz & Cianciara [45], we assume that the key factors of human health are environmental NGOs, economic development, technology development, and health expenditure. We add the environmental NGOs and initiate with the following econometric models:

$${Health}_{t}=\pi_{0}+\pi_{1}E{NGOs}_{t}+\pi_{2}{GDP}_{t}+\pi_{3}{TECH}_{t}+\pi_{3}{HE}_{t}+\varepsilon_{t}$$ (1)

Eq (1) is human health (Health) model that depends on environmental NGOs (ENGOs), GDP per capita (GDP), technology development (TECH), and health expenditure (HE). The rise in environmental NGOs improves health by reducing pollution emissions, thus estimates of π_1_ should be positive. Economic development, technology development, and health spending have a positive effect on health outcomes, thus an estimate of π_2_, π_3_, π_5_ could be positive. The estimates discussed above are long-run effects. Regarding short-run effects, Eq (1) should be converted to an error-correction framework.

$${\Delta Health}_{t}=\pi_{0}+\sum_{k=1}^{q}\beta_{1k}{\Delta Health}_{t-k}+\sum_{k=0}^{n}\beta_{2k}{\Delta ENGOs}_{t-k}+\sum_{k=1}^{n}\beta_{3k}{\Delta GDP}_{t-k}+\sum_{k=0}^{n}\beta_{4k}{\Delta TECH}_{t-k}+\sum_{k=0}^{n}\beta_{5k}{\Delta HE}_{t-k}++\pi_{1}{Health}_{t-1}+\pi_{2}{ENGOs}_{t-1}+\pi_{3}{GDP}_{t-1}+\pi_{4}{TECH}_{t-1}+\pi_{5}{HE}_{t-1}+\lambda .{ECM}_{t-1}+\varepsilon_{t}$$ (2)

Eq (2) includes short-term parameters (β) and long-term parameters (π) in error-correction models. The ARDL method is a workhorse in economy-specific empirical analysis. Regarding cointegration, Pesaran et al. [46] recommend F-test and ECM tests for the meaningfulness of estimates. Eq (2) signifies the ‘‘unrestricted error-correction model”. This method has some gains over other methods [47]. First, it is comparatively better in a small sample. Second, if the model variables are I(1), I(0), or mixed, then ARDL can be applied. For stationarity, the study used DF-GLS and Phillips and Perron (PP) unit root tests and found there is a mixed result. The ARDL method is more useful for estimating short-run and long-run effects in a single equation [10]. The ARDL is ineffective if model variables occur I(2) or are higher-order. For post-estimations, to test the autocorrelation problem, we have applied the LM test. The model’s correct specifications are identified by using the RESET test. Finally, to judge the stability of estimates, we have applied the CUSUM tests.

This study is exploring the nexus between environmental NGOs and human health in China over the period 1995 to 2020. Three proxies are used to measure human health. These are life expectancy (LE), infant mortality rate (IMR), and death rate (DR). The data for these three measures is assembled from the WDI. The data for the total number of environmental NGOs is attained from the Environmental Protection Bureaus (EPB). Besides these variables, technology, GDP per capita, and health expenditures are used as control variables. Liu et al. [48] study highlights that technology is an important factor that can influence a country’s economic growth and overall well-being. Technology intensity is determined through total patent applications. Ozturk & Ullah [49] reveals that GDP per capita is an important measure of a country’s economic development and standard of living. Countries with higher GDP per capita tend to have higher levels of human development and better living standards. GDP per capita is measured as constant at 2010 US$. Countries with higher health expenditures tend to have better healthcare outcomes, such as lower rates of mortality and longer life expectancy. Health expenditures are taken as % of GDP. The data for these three control variables is attained from the WDI. A data summary is given in Table 1. The mean values are found as: 2.736 for IMR, 4.299 for LE, 6.813 for DR, 12.72 for ENGO, 8.352 for GDP, 12.32 for TECH, and 4.393 for HE. Maximum and minimum values are reported as: for IMR (Max: 3.648; Min: 1.705), for LE (Max: 4.345; Min: 4.247), for DR (Max: 7.14; Min: 6.4), for ENGO (Max: 13.33; Min: 11.86), for GDP (Max: 9.246; Min: 7.326), for TECH (Max: 14.24; Min: 9.836), and for HE (Max: 5.35; Min: 3.675). The standard deviations are reported as follows: 0.628 for IMR, 0.031 for LE, 0.305 for DR, 0.474 for ENGO, 0.632 for GDP, 1.447 for TECH, and 0.495 for HE. The skewness test reports that all series are negatively skewed except HE, which is positively skewed. The Kurtosis estimates are quite satisfactory.

**Table 1:** List of the variables and descriptive statistics.

Variables	Definitions	Mean	Median	Max	Min	Std. Dev.	Skewness	Kurtosis	Sources
IMR	Mortality rate, infant (per 1,000 live births)	2.736	2.740	3.648	1.705	0.628	-0.082	1.694	WDI
LE	Life expectancy at birth, total (years)	4.299	4.300	4.345	4.247	0.031	-0.115	1.786	WDI
DR	Death rate, crude (per 1,000 people)	6.813	6.985	7.14	6.4	0.305	-0.247	1.209	WDI
ENGO	Total number of environmental NGOs	12.72	12.88	13.33	11.86	0.474	-0.435	1.782	EPB
GDP	GDP per capita (constant 2015 US$)	8.352	8.414	9.246	7.326	0.632	-0.118	1.617	WDI
TECH	Patent applications, total (residents and nonresidents)	12.32	12.49	14.24	9.836	1.447	-0.229	1.761	WDI
HE	Current health expenditure (% of GDP)	4.393	4.346	5.35	3.675	0.495	0.283	1.971	WDI

### Empirical results

Before regression analysis, this study employed the unit root tests of the data. Thus, Table 2 described the results of DF-GLS, PP, and ZA unit root tests. The unit root test findings revealed that only GDP is a level stationary variable, whereas all other variables such as IMR, DR, LE, ENGO, TECH, and HE are stationary at first difference. The outcomes of all these three unit root tests (i-e DF-GLS, PP, and ZA) confirmed that the study may apply the ARDL technique. We have used three proxy measures of health outcome in our model. These are life expectancy, infant mortality rate, and death rate. Infant mortality rate and death rate are used to check the robustness of findings.

**Table 2:** Unit root tests results.

	DF-GLS		PP		ZA			
	I(0)	I(1)	I(0)	I(1)	I(0)	Break date	I(1)	Break date
IMR	-1.023	-3.325**	1.635	-2.689*	2.824	2013	-4.365*	2019
DR	-1.345	-2.654**	-0.875	-2.745*	-2.321	2005	-6.356***	2006
LE	-0.231	-3.325**	-0.187	-3.245***	-3.356	1998	-4.658**	2015
ENGO	-0.954	-3.321**	-0.562	-2.745*	-5.321***	2000		
GDP	-1.902*		-2.805*		-6.356***	2004		
TECH	-0.754	-4.325***	-2.452	-4.562***	-2.965	2010	-5.658***	2018
HE	-0.721	-4.895***	-1.023	-4.652***	-2.785	2011	-5.654***	2007

**Note:** Critical values of ADF, DF-GLS, and ZA tests at 5% level are -2.991, -1.955, and -4.443.

***p<0.01

**p<0.05; and

*p<0.1.

Table 3 depicts the ARDL estimates findings. The coefficient estimates show that ENGO has a negative impact on IMR and infers that a 1% increase in ENGO has 0.264% decrease in IMR in the long run. The coefficient of ENGO confirms that 1% increase ENGO has a 0.284% decrease in DR in the long run. The long run and short run empirical findings disclosed that ENGO has a positive impact on LE. This infers that 1% increase in ENGO moves to a 0.005% increase in LE in the long run and 0.009% increase in LE in the short run. Our findings suggest that ENGOs play a positive and significant role in improving health outcomes in China. Since climate change and global warming are significant contributors to worldwide health problems, therefore improving environmental quality can significantly improve the health status in China. ENGOs now have an even more positive state to participate actively in China’s environmental policies. Due to the Chinese central government’s current efforts for social solidarity, or formally "a harmonious society," officials at all tiers have been pushed to become increasingly helpful to NGOs to support impoverished populations and minimize social tensions and health concerns [50]. Among the major factors in increasing good governance and decentralizing public governance is a sign of healthy liberal democracy [51]. ENGOs have played a significant role in global advocacy systems and international environmental negotiations [52]. Therefore, the positive role of ENGOs in improving health-related indicators in China is not surprising because ENGOs improve environmental quality by reducing CO2 emissions, which will help eliminate many health concerns [19,51].

**Table 3:** Long and short-run coefficient estimates.

	IMR		DR		LE	
	Coeff.	t-Stat	Coeff.	t-Stat	Coeff.	t-Stat
**Short-run**						
ENGO	-0.012	-0.895	-0.052	-0.412	0.009**	1.964
ENGO(-1)			-0.214	-1.556	0.004*	1.834
ENGO(-2)					0.002***	3.567
GDP	-0.008	-0.240	-0.864***	-2.886	0.010**	2.355
GDP(-1)					0.004	1.066
GDP(-2)					0.002	1.222
TECH	-0.017**	-2.137	-0.103	-1.252	0.011***	4.020
HE	-0.009**	-2.131	-0.201***	-3.147	0.008***	3.011
HE(-1)			-0.177***	-3.013	0.004	0.929
**Long-run**						
ENGO	-0.264***	-2.874	-0.284*	-1.784	0.005***	3.485
GDP	-0.166*	-1.919	-1.508***	-4.484	0.049***	3.301
TECH	-0.365*	-1.823	-0.179*	-1.824	0.004***	3.654
HE	-0.202**	-2.204	-0.659***	-6.594	0.001*	1.862
C	-9.723***	-4.018	-2.921**	-2.056	3.859***	7.521
**Diagnostics**						
F-test	5.698***		6.102***		12.32***	
ECM(-1)*	-0.474***	-6.322	-0.573***	-6.987	-0.478***	-5.152
LM	1.365		1.589		2.012	
RESET	0.325		1.023		0.658	
CUSUM	S		S		S	
CUSUM-sq	S		S		S	

**Note**:

***p<0.01

**p<0.05; and

*p<0.1.

In support of our findings, various studies argue that ENGOs can play an important role in protecting human health by advocating for policies and practices that support a healthy environment. For instance, Chen et al. [38] describes that environmental NGOs may engage in direct interventions to reduce or prevent environmental harm, such as through pollution monitoring, community organizing, and advocacy for stronger environmental regulations. These interventions can help to reduce the exposure of people to harmful pollutants, which in turn can improve their health. Tu et al. [37] argues that environmental NGOs can also play a critical role in educating the public about the health impacts of environmental degradation and pollution. By raising awareness of these issues, they can help to promote individual behavior change and mobilize communities to take action to protect the environment and their health. Another study done by Patrick et al. [53] reveals that environmental NGOs can also partner with other organizations and institutions to promote healthy environments and protect human health. For example, they may collaborate with public health organizations to develop joint initiatives that address environmental health issues, or partner with community groups to develop local environmental improvement projects.

Regarding control variables, finding show that has a negative influence on IMR and DR. This implies that IMR and DR decrease by GDP. The coefficient of GDP revealed as a 1% increase in GDP causes to (0.166%) 1.508% decrease in (IMR) DR in the long run. The coefficient of GDP also revealed that 1% increase in GDP leads to a 0.049% increase in LE in the long run. Similarly, TECH has a negative effect on IMR that confirms that the infant mortality rate decreases by technology progress. The coefficient of TECH revealed as a 1% increase in TECH causes to 0.365% decrease in IMR in the long run. The coefficient estimate of TECH noted that a 1% increase TECH has a 0.179% decrease in DR in the long run, which shows that technology development decreases the death rate decreases. The coefficient of TECH also depicted that a 1% increase in TECH leads to a 0.004% increase in LE in the long run.

Moreover, health expenditures, technological improvement, and affluence also positively contribute to uplifting the health status of Chinese people. The positive relationship between health expenditures and health outcomes is supported by Panopoulou & Pantelidis [54] and Arthur & Oaikhenan [55]. This is not surprising because the increased health expenditure can help improve the quantity and quality of health-related infrastructures such as hospitals, laboratories, medical colleges, and paramedics staff. Similarly, technological advancement in China can help improve healthcare services because services that engage with goods, data, technology, and individuals, including those in the healthcare system, may all benefit from technological innovation [56]. Lastly, the rise in the national income of China can provide more financial resources for the development of health infrastructure and state-of-the-art healthcare services [57].

However, HE has a negative impact on IMR and DR in both long run and short run which means that health expenditures reduce significantly the infant mortality rate and death rate. The coefficient of HE reported that a 1% increase HE results to 0.202% and 0.659% decrease in IMR and DR in long run. while 0.009% decrease in IMR along with 0.201% decrease in DR in short run. However, the coefficient of HE has a positive impact on LE and infers that 1% increase in HE increases LE by 0.001% increase in long run and an increase in LE by 0.008% in short run.

Lastly, a few diagnostic tests are also applied such as F-test and ECM (-1) that verify the long run cointegration amongst all variables in all models. The convergence tendency towards long-run equilibrium in all three models is validated by the negative sign attached to ECM. The LM test results confirmed that there is no problem of serial correlation. The correct specification of models are confirmed through the Ramsey RESET test. The CUSUM test results verified the stability condition. The causality test results are shown in Table 4. There is a bidirectional causal association among ENGO and IMR and ENGO and LE, whereas the unidirectional causal connection runs from ENGO to DR.

**Table 4:** Causality test results.

Null Hypothesis:	F-Stat	Prob.	Null Hypothesis:	F-Stat	Prob.	Null Hypothesis:	F-Stat	Prob.
ENGO →IMR	4.295	0.026	ENGO →DR	3.507	0.051	ENGO →LE	5.846	0.011
IMR →ENGO	12.09	0.000	DR →ENGO	0.026	0.974	LE →ENGO	5.761	0.011
GDP → IMR	1.494	0.250	GDP →DR	7.894	0.003	GDP →LE	17.86	0.000
IMR → GDP	6.495	0.007	DR →GDP	1.837	0.187	LE →GDP	3.483	0.051
TECH →IMR	3.752	0.042	TECH →DR	1.930	0.173	TECH →LE	2.104	0.149
IMR →TECH	0.170	0.845	DR →TECH	0.353	0.707	LE →TECH	2.436	0.114
HE →IMR	0.757	0.483	HE →DR	0.850	0.443	HE →LE	7.339	0.004
IMR →HE	2.495	0.109	DR →HE	5.901	0.010	LE →HE	1.408	0.269
GDP →ENGO	11.57	0.001	GDP →ENGO	11.57	0.001	GDP →ENGO	11.57	0.001
ENGO →GDP	0.560	0.581	ENGO →GDP	0.560	0.581	ENGO →GDP	0.560	0.581
TECH →ENGO	9.473	0.001	TECH →ENGO	9.473	0.001	TECH →ENGO	9.473	0.001
ENGO →TECH	1.978	0.166	ENGO →TECH	1.978	0.166	ENGO →TECH	1.978	0.166
HE →ENGO	2.527	0.106	HE →ENGO	2.527	0.106	HE →ENGO	2.527	0.106
ENGO →HE	1.656	0.217	ENGO →HE	1.656	0.217	ENGO →HE	1.656	0.217
TECH →GDP	4.292	0.029	TECH →GDP	4.292	0.029	TECH →GDP	4.292	0.029
GDP →TECH	1.856	0.184	GDP →TECH	1.856	0.184	GDP →TECH	1.856	0.184
HE →GDP	0.242	0.787	HE →GDP	0.242	0.787	HE →GDP	0.242	0.787
GDP →HE	11.26	0.001	GDP →HE	11.26	0.001	GDP →HE	11.26	0.001
HE →TECH	0.123	0.885	HE →TECH	0.123	0.885	HE →TECH	0.123	0.885
TECH →HE	1.237	0.313	TECH →HE	1.237	0.313	TECH →HE	1.237	0.313

## Conclusion and implications

Today’s most pressing issues are climate change and global warming, which severely influence people’s health and affluence. The IPCC 2018 warns that rising CO2 emissions bring "major environmental problems" sooner than expected, negatively impacting people’s physical and mental health worldwide. This has irked the general public, civil society, and world leaders, and they have started focusing on the factors that can help diminish carbon footprints and subsequent climate change and health concerns. In this regard, the role of non-profit organizations is on the rise because such organizations are exerting pressure on world leaders to control the harmful impacts of climate change by restricting CO2 emissions. Recently, the emergence of environmental NGOs has proved beneficial in improving environmental quality and related health issues; however, not much empirical evidence is available that has investigated the relationship between the role of ENGOs and human health. To fill the gap mentioned above, this study attempts to investigate the impact of ENGOs on human health in China.

We start our empirical analysis by first checking the stationary properties of the variables by employing DF-GLS, PP, and ZA unit root tests. These test findings imply that variables included in the analysis are a mixture of I(0) and I(1), which induces us to use the ARDL model. The ARDL model results demonstrate that the long-run impact of ENGO is negative on infant mortality and death rate, meaning that an increase in the proportion of ENGOs in China considerably decreases infant mortality and death rate. On the other hand, ENGOs have a favorable influence on life expectancy in China, demonstrating ENGOs’ supporting role in raising birth life expectancy. In the short run, estimates of ENGOs have no substantial influence on newborn mortality and death rates in China, whereas ENGOs have a positive and significant impact on life expectancy. These results imply that ENGOs help improves people’s health status in China, which is also supported by the rise in GDP, technology, and health expenditures. The causal analysis confirms the bi-directional causal link between ENGO and IMR and ENGO and LE, while the uni-directional causal link runs from ENGO to DR.

We can make some crucial policy recommendations based on the findings. The study’s findings validated favorable influence of ENGOs on human health in China. Environmental NGOs must assist the government in increasing public knowledge about environmental issues. The authorities may inspire people and companies to adopt pro-environmental behaviors with the assistance of social movements supported and organized by the ENGOs. Along with the increase in the number of ENGOs, the environment improves, which also improves people’s health status in China. Governments should consider increasing funding for environmental health research to support the work of these organizations. Environmental NGOs often advocate for stronger environmental regulations to protect human health. Governments should take these recommendations seriously and work to enact and enforce regulations that limit exposure to harmful pollutants and chemicals. Environmental NGOs can play a critical role in raising public awareness about the health impacts of environmental degradation and pollution. Governments should work with these organizations to ensure that the public is educated about the potential health risks associated with exposure to environmental toxins. Governments should work to establish collaborative partnerships with environmental NGOs to address environmental health issues. This could include joint research projects, public awareness campaigns, and other initiatives designed to protect public health. Governments should work to ensure that individuals have the ability to hold polluters accountable for the harm they cause. Moreover, technological improvement also has a positive impact on the health status of people. Therefore, policymakers must invest in research and development activities in the health sector that would help improve health-related technologies and equipment. Further, to improve health facilities and infrastructure, the government of China needs to increase health spending. The increase in health spending can also be used to provide health insurance to a deprived faction of society.

Our study acknowledged a few limitations and provides important future directions. Our study focuses on the Chinese economy at the aggregate level. Due to the unavailability of environmental NGOs data for provinces, we could not include provinces of China in our sample. Future research should expand analysis for provinces of China after collecting the required data for Chinese provinces. Environmental NGOs play a critical role in promoting environmental protection and improving access to clean water and air, which may have indirect effects on human health. Future research should continue to explore the specific impact of these organizations on health outcomes and the mechanisms through which they operate. Future studies should also investigate the effectiveness of different strategies used by environmental NGOs in addressing environmental problems and their potential to reduce environmental risks and improve public health.

## Supporting information

S1 Dataset (XLSX)
